# MRI‐visible dilated perivascular spaces in healthy young adults: A twin heritability study

**DOI:** 10.1002/hbm.25194

**Published:** 2020-09-08

**Authors:** Yangsean Choi, Yoonho Nam, Yera Choi, Jiwoong Kim, Jinhee Jang, Kook Jin Ahn, Bum‐soo Kim, Na‐Young Shin

**Affiliations:** ^1^ Department of Radiology, Seoul Saint Mary's Hospital, College of Medicine The Catholic University of Korea Seoul Republic of Korea; ^2^ Division of Biomedical Engineering Hankuk University of Foreign Studies Yongin‐Si Republic of Korea

**Keywords:** convolutional neural network, heritability, MRI, perivascular spaces, twin study

## Abstract

We investigated the narrow‐sense heritability of MRI‐visible dilated perivascular spaces (dPVS) in healthy young adult twins and nontwin siblings (138 monozygotic, 79 dizygotic twin pairs, and 133 nontwin sibling pairs; 28.7 ± 3.6 years) from the Human Connectome Project. dPVS volumes within basal ganglia (BGdPVS) and white matter (WMdPVS) were automatically calculated on three‐dimensional T2‐weighted MRI. In univariate analysis, heritability estimates of BGdPVS and WMdPVS after age and sex adjustment were 65.8% and 90.2%. In bivariate analysis, both BGdPVS and WMdPVS showed low to moderate genetic correlations (.30–.43) but high shared heritabilities (71.8–99.9%) with corresponding regional volumes, intracranial volumes, and other regional dPVS volumes. Older age was significantly associated with larger dPVS volume in both regions even after adjusting for clinical and volumetric variables, while blood pressure was not associated with dPVS volume although there was weak genetic correlation. dPVS volume, particularly WMdPVS, was highly heritable in healthy young adults, adding evidence of a substantial genetic contribution in dPVS development and differential effect by location. Age affects dPVS volume even in young adults, while blood pressure might have limited role in dPVS development in its normal range.

## Graphical abstract

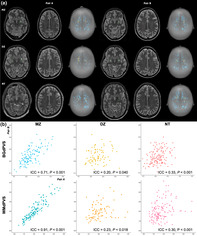

## 1 INTRODUCTION

Perivascular spaces (PVS), also known as Virchow–Robin spaces, are normal fluid‐filled cavities surrounding small cerebral penetrating vessels and are known to be an important component of the glymphatic system—a recently discovered system responsible for clearing waste and interstitial fluid from the brain, delivering energy substrates and maintaining the immune system (Jessen, Munk, Lundgaard, & Nedergaard, 2015; Tarasoff‐Conway et al., 2015). As PVS are normally microscopic, they are usually considered enlarged or dilated when visible on MRI (Groeschel, Chong, Surtees, & Hanefeld, 2006). Since recent studies have reported associations between dilated MRI‐visible PVS (dPVS) and aging, hypertension, small vessel disease (Y. C. Zhu et al., 2010), Alzheimer's disease (Banerjee et al., 2017), and multiple sclerosis (Wuerfel et al., 2008), there has been growing interest in defining the clinical implications and risk factors of dPVS.

dPVS are mainly found in the basal ganglia (BG) along the path of the lenticulostriate arteries and in the subcortical white matter (WM) along the path of the perforating medullary arteries (Kwee & Kwee, 2007). dPVS have distinct characteristics based on their location. dPVS in BG (BGdPVS) are associated with aging, hypertension (Martinez‐Ramirez et al., 2013), imaging features of hypertensive angiopathy (i.e., WM hyperintensity, lacunes, and deep microbleeds; Andreas Charidimou et al., 2017; Duperron et al., 2018; Martinez‐Ramirez et al., 2013), and subcortical vascular cognitive impairment (Banerjee et al., 2017). dPVS in WM (WMdPVS), on the other hand, are linked to cortical amyloid‐ß deposition, apolipoprotein E ε4 allele presence (Roher et al., 2003), cerebral amyloid angiopathy (A. Charidimou et al., 2013; Andreas Charidimou et al., 2017; Martinez‐Ramirez et al., 2013) and its associated imaging features such as lobar hemorrhage, lobar microbleeds, and cortical superficial siderosis, and Alzheimer's disease (Banerjee et al., 2017). These differences raise the possibility of there being a distinct underlying pathophysiology behind the development of dPVS according to location.

To understand the pathophysiology of dPVS and to define degree of influence by modifiable environmental determinants for PVS dilatation, knowledge of the possible genetic contribution for PVS dilatation would be helpful by assessing its heritability, which is defined as the proportion of variation observed within a phenotypic trait that is due to genetic variation. To date, only one study has investigated the heritability of dPVS with genome‐wide genotyping in a large population‐based elderly cohort (Duperron et al., 2018). The authors of this previous study found differential heritability patterns between BGdPVS and WMdPVS, in which higher heritability was seen in WMdPVS than that in BGdPVS (79 vs. 49%) and the overlap of genetic effects between the two phenotypes, in other words, genetic correlation, was larger with WM hyperintensities in BGdPVS than in WMdPVS.

However, this past study was performed with an elderly population (mean age 72.8 years) and even though the study population had no history of stroke and dementia, it had a high prevalence of hypertension (76.6%) which is a well‐known risk factor for dPVS (Duperron et al., 2018). Furthermore, dPVS was assessed with a subjective 4‐grade scale, and this type of assessment is time‐consuming and susceptible to inevitable intra‐/inter‐rater variability.

As heritability has a dynamic nature that is influenced by changes in gene expression and environmental exposure as well as various genetic‐environmental interactions over time (Bergen, Gardner, & Kendler, 2007; Eaves, Long, & Heath, 1986), it is also important to understand that heritability in elderly people might be more influenced by environmental factors accumulated over a longer period of time than in young healthy adults. Moreover, a high prevalence of vascular risk factors such as high blood pressure (BP) and hypercholesterolemia could affect heritability estimates (Eaves et al., 1986).

Therefore, our main goal was to investigate the heritability of PVS dilatation in healthy young adults objectively using a fully automated dPVS quantification method. In doing so, we used the Human Connectome Project (HCP) dataset which consisted of twins and nontwin siblings with various clinical information and high‐resolution three‐dimensional (3D) structural MRI available.

## 2 METHODS

This retrospective study was approved by our institutional review board. The requirement for informed consent was waived as a publicly available dataset was used.

### 2.1 Subjects

Heritability analysis was performed using structural images and demographic data included in the WU‐Minn Human Connectome Project dataset which recruited 1,200 healthy adult twins and nontwin siblings 22–35 years old to characterize the relationships between brain circuits, behavior, and genetics (Van Essen et al., 2013).

Among 1,206 individuals available in the March 2017 (S1200) release, 1,113 individuals who had 0.7 mm isotropic 3D T1‐ and T2‐weighted images obtained on 3 T MRI were initially screened. Individuals with the following criteria were excluded: (a) severe neurodevelopmental, documented neuropsychiatric, or neurological disorders; (b) high BP, diabetes, or significant cardiovascular disease; (c) born preterm—defined as born before 34th weeks of gestation for twins and before 37 weeks of gestation for nontwins; (d) zygosity not verified by genotyping; and (e) nontwin siblings without their respective pairs or with different biological fathers or mothers. Detailed information about recruitment—as well as the inclusion and exclusion criteria—of the Human Connectome Project is described in a previous study (Van Essen et al., 2013). For this study, a total of 700 subjects (350 twin or nontwin sibling pairs), consisting of 138 monozygotic (MZ) twin pairs, 79 dizygotic (DZ) twin pairs, and 133 nontwin sibling pairs, were finally included in the heritability analysis.

To assess the reliability of the automated dPVS segmentation method, 45 subjects who underwent two MRI acquisitions on different dates (mean interval of 134.8 days [range, 18–328 days]) were also included from the WU‐Minn Human Connectome Project Retest dataset.

### 2.2 Imaging acquisition

All MRI data were obtained at Washington University in St. Louis, MO, on a Siemens 3T MR scanner “Connectome Skyra,” which was customized with a 100mT/m gradient coil and inner bore diameter of 56 cm, using a standard 32‐channel head coil.

A 3D T1‐weighted Magnetization‐Prepared Rapid Acquisition with Gradient Echo (MPRAGE) sequence was acquired with the following parameters: sagittal acquisition with FOV = 224 × 224 × 180 mm; voxel size = 0.7 × 0.7 × 0.7 mm^3^; repetition time = 2,400 ms; echo time = 2.14 ms; inversion time = 1,000 ms; band width = 210 Hz/pixel; flip angle = 8°; GeneRalized Autocalibrating Partial Parallel Acquisition (GRAPPA) factor = 2; and total acquisition time = 7 min 40 s.

A 3D T2‐weighted Sampling Perfection with Application‐optimized Contrasts using different flip angle Evolution (SPACE) sequences was acquired with the following parameters: sagittal acquisition with field of view = 224 × 224 × 180 mm; voxel size = 0.7 × 0.7 × 0.7 mm^3^; repetition time = 3,200 ms; echo time = 565 ms; echo spacing = 3.53 ms; turbo factor = 314; echo train duration = 1,105 ms; band width = 744 Hz/pixel; variable flip angle; GRAPPA factor = 2; and total acquisition time = 8 min 24 s.

More detailed information regarding imaging protocols can be found in the WU‐Minn Human Connectome Project S1200 Release Reference Manual (Van Essen et al., 2013).

### 2.3 Automated dPVS segmentation method

When measuring dPVS volume, 3D T1‐ and T2‐weighted images were used along with Freesurfer generated sub‐region masks from the Human Connectome Project database. dPVS volumes were calculated fully automatically through the following two steps:

#### 2.3.1 Potential dPVS voxel extraction from T2‐weighted images

Potential dPVS voxels were extracted from T2‐weighted images using 3D Frangi filtering (Frangi, Niessen, Vincken, & Viergever, 1998), which calculates the vesselness measure based on the eigenvalues of the Hessian. This method is known to be particularly useful for delineating enhancing vascular structures (Jimenez‐Carretero, Santos, Kerkstra, Rudyanto, & Ledesma‐Carbayo, 2013; Xiao et al., 2011) and also for detecting dPVS on medical images (Ballerini et al., 2016; Zong, Park, Shen, & Lin, 2016). To reduce the sensitivity of the signal intensity variations across subjects, the signal intensity of the T2‐weighted image was normalized by subtracting the mean of the signal intensities, and then divided by the standard deviation of signal intensities before the Frangi filter was applied. After normalizing signal intensity, to calculate the vesselness measure, a 3D Frangi filter was applied to the T2‐weighted image and thresholding was performed to extract potential dPVS voxels. The processed T2‐weighted images had a large number of false positives outside the brain region. To reduce these false positives, the WM and BG masks from the Freesurfer segmentation results were used.

#### 2.3.2 Refinement by convolutional neural network‐based classification

**FIGURE 1:**
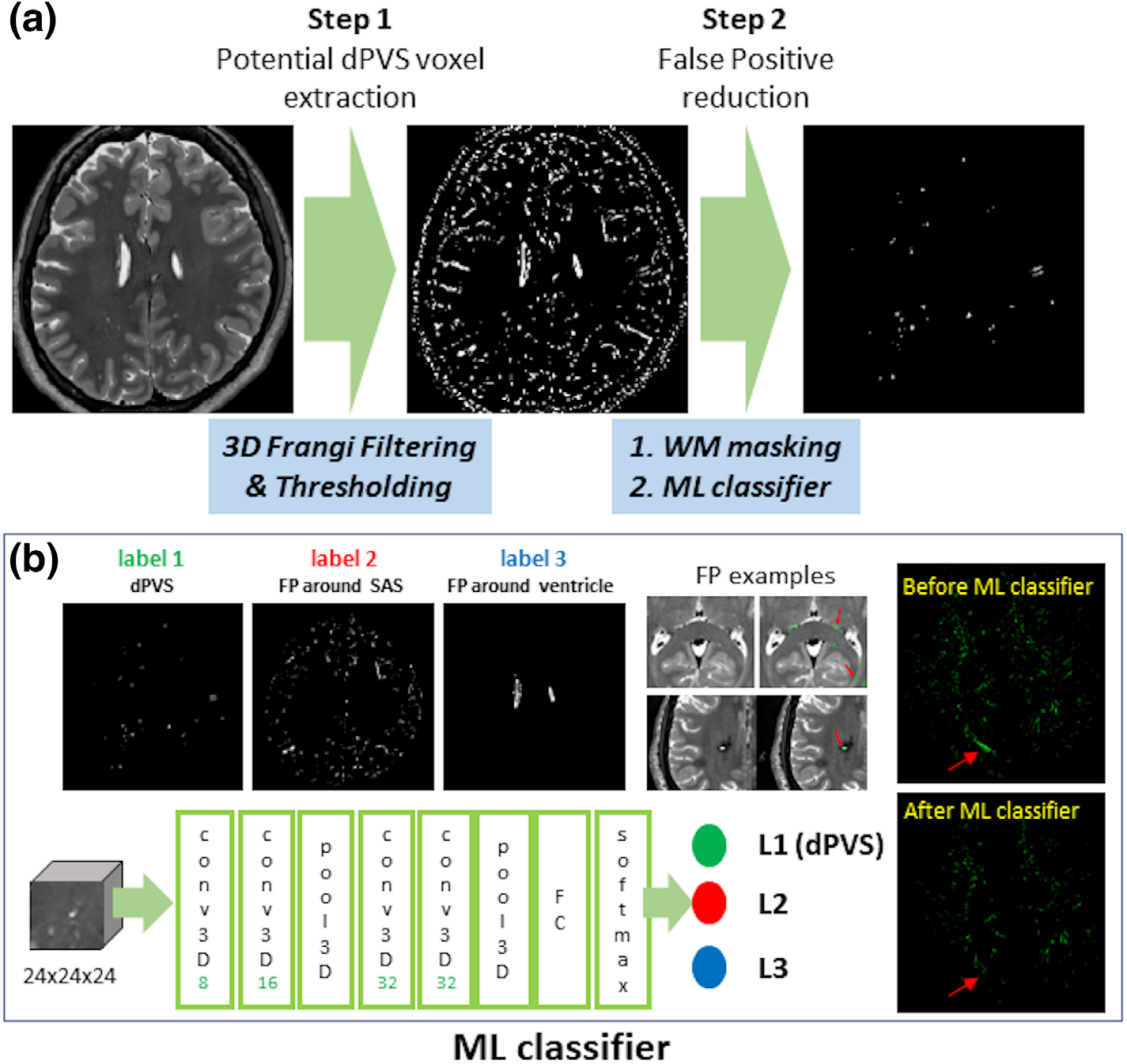
Workflow diagram of fully automated dilated perivascular space (dPVS) segmentation. (a) Extraction of potential dPVS voxels using three‐dimensional Frangi filtering and thresholding with subsequent false‐positive reduction via white matter (WM) masking, and (b) three‐dimensional convolutional neural network (CNN) machine learning classifiers with a 24 × 24 × 24 three‐dimensional input data patch. FP, false positives; ML, machine‐learning; SAS, subarachnoid space

To reduce the remaining false positives around the ventricles or subarachnoid space, we trained a 3D deep convolutional neural network (CNN) to classify three classes (Class 1: dPVS, Class 2: false positives around the ventricles, and Class 3: false positives around the subarachnoid space) using a 24 × 24 × 24 3D patch as input data as described in Figure 1.

A kernel size for 3D convolution was 3 × 3 × 3 for all convolutional layers and the number of nodes for the fully connected layer was 128. A rectified linear unit activation was applied after each convolution layer and 3D max‐pooling (2 × 2 × 2) was performed after two consecutive convolutional layers. The number of channels for the convolutional layers was 8, 16, 32, and 32 in order. A cross‐entropy loss between the network output and the label was used to train the network. For this purpose, three‐class labeling on the processed images in Section 2.3.1. was manually performed for 20 randomly selected nontwin subjects. To increase the robustness of the input variations, several data augmentation steps such as flipping, adding randomly generated noises or biases, and multiplying randomly generated scaling factors were applied for each patch data. To reduce the effects of the unbalanced numbers between individual classes, each class was fed to the network at uniform frequency in the training process. The trained network was used to generate the final dPVS voxels from the potential dPVS voxels for the test dataset. Based on the output of the 3D CNN algorithm, regional dPVS voxels were counted from the BG and WM segmented masks. The volume of dPVS was calculated by the product of the number of dPVS voxels and volume of 1 voxel (i.e., 0.7 × 0.7 × 0.7 = 0.343 mm^3^).

### 2.4 Statistical analysis

#### 2.4.1 Reliability assessment of the automated dPVS segmentation method

The reliability of the automated dPVS segmentation method was assessed using the intraclass correlation coefficient (ICC) for the automatically measured dPVS volume in 45 subjects with test and retest datasets.

#### 2.4.2 Validity assessment of the automated dPVS segmentation method

For validity assessment, fractions of false‐positive and false‐negative volumes from the automated dPVS segmentation method were compared with those from manual segmentation according to the STandards for ReportIng Vascular changes on nEuroimaging (STRIVE) criteria (Wardlaw, Smith, Biessels, et al., 2013). Axial T2‐weighted images of 10 randomly chosen subjects were visually assessed by two board‐certified radiologists (BLINDED and BLINDED with 6 and 13 years of experience in neuroradiology, respectively) who were blinded to the volumetric results of the automated dPVS segmentation method. dPVS were defined as lesions showing high signal intensity on T2‐weighted images similar to that of cerebrospinal fluid and following the course of penetrating vessels with a diameter of less than 3 mm. dPVS in BG and WM were manually segmented on the axial images and their volumes were calculated at the level of anterior commissure and at the upper end of corpus callosum, respectively, first independently and then in consensus by the two neuroradiologists. ICCs between the manually and automatically measured dPVS volumes were also calculated.

#### 2.4.3 Group comparisons of clinical and volumetric variables and their association with dPVS volumes

According to the results of the normality tests, the clinical and volumetric variables of each group were demonstrated as medians (interquartile ranges) for continuous variables and numbers (percentages) for categorical variables. The continuous and categorical variables were compared among groups using linear and generalized linear mixed models, respectively, thereby accounting for the within‐pair phenotypic correlations. In these models, subject group was set as a fixed effect while twin pair (i.e., Family ID provided by Human Connectome Project dataset) was set as a random effect. P values for pair‐wise group comparisons were corrected for multiple comparisons using the Bonferroni method. To explore possible genetic influences, within‐pair differences of clinical and volumetric variables were compared between groups via the Kruskal–Wallis test with *posthoc* Tukey's test for continuous variables and the Chi‐squared test with Bonferroni correction for categorical variables. To explore factors that may possibly affect dPVS volume and adjust for their effects on the following heritability analyses, linear mixed models were used for BGdPVS and WMdPVS with each variable set as a fixed effect and twin pair (i.e., Family IDs provided by Human Connectome Project dataset) set as a random effect. Age and sex were included as covariates in these models except when analyzing the variables of age and sex themselves. For a simplified statistical analysis, we dichotomized ethnic groups into White and nonWhite, with the latter group including Black or African American and Asian, natural Hawaiian, or other Pacific Islanders. Subjects who did not know or report their ethnic group or belonged to more than one ethnic group were excluded when the ethnic group was analyzed as a covariate.

#### 2.4.4 Heritability analysis using univariate and bivariate models

We implemented the classical twin study design, in which narrow‐sense heritability (*h* ^2^) was defined as the ratio of variance from a phenotypic measurement, which can be shown as additive genetic variation [A]/total observed variation (genetics [A] + shared environment [C] + unique environment [E]). Since the common or shared environment is considered to be the same within a family, [C] was set as 1 for all twins and nontwin siblings (Patel et al., 2017; Schneider et al., 2019). The genetic variation [A] was set as 1 for MZ twin pairs and 0.5 for DZ twins and nontwin siblings based on previous literature assuming that DZ twins and nontwin siblings share on average 50% of their genes (Patel et al., 2017, 2018). Prior to analysis, age, pulse pressure (systolic BP‐diastolic BP), BGdPVS, WMdPVS, BG, WM volumes, and intracranial volumes (ICV) were standardized to z‐scores. Pulse pressure was included in the heritability analysis since the pulsatile component rather than the static component of BP was reported to be associated with dPVS (Del Brutto & Mera, 2018). Narrow‐sense heritability was estimated for BGdPVS and WMdPVS volumes in univariate and bivariate analyses.

In the univariate analysis, both BGdPVS and WMdPVS volumes were first adjusted for age and sex, hereafter named as *unadjusted*. In addition to the adjustment for age and sex, the volumes were sequentially adjusted for (a) regional volume (BG volume for BGdPVS and WM volume for WMdPVS) or (b) ICV—termed as *partially adjusted*. For *completely adjusted* univariate models, age, sex, regional volumes, and ICV were adjusted. In addition to these covariates, pulse pressure and ethnic group were also included as additional covariates to assess their influence. We assessed variable inflation factors to ensure that there was no issue with multicollinearity in our models.

In the bivariate analysis, after adjusting for age and sex, genetic correlation and shared heritability of dPVS volumes with pulse pressure, regional volume, ICV, and counterpart dPVS (BGdPVS to WMdPVS and vice versa) were calculated, thereby accounting for the shared genetic variation between the two phenotypes. Genetic correlation represents the measurement of genetic overlap between two traits, which indicates the similarity of the genetic variation between two phenotypes. Genetic correlation ranges from −1 to 1, with a correlation of 1 implying that the two traits in question are influenced by the same genetic factors, while a correlation of 0 represents independent genetic effects. Shared heritability assesses the amount of genetic variation shared between two traits; that is, the contribution of the shared genetic effect to the observed phenotypic correlation. The Cholesky decomposition ACE models enable the calculation of genetic correlation and shared heritability.

From the models, we could obtain three paths each for genetics, shared environment, and unique environment: *a*_11_ denoted the path from the first latent genetic variable to the first trait, *a*_12_ the path from the first latent genetic variable to the second trait, and *a*_22_ the path from the second latent genetic variable to the second trait; *c*_11_, *c*_12_, and *c*_22_ denoted the shared environment and *e*_11_, *e*_12_, and *e*_22_ the unique environment. Genetic correlation and shared heritability were then calculated as:

$$\text{genetic correlation}=\frac{a_{12}}{\sqrt{{a_{12}}^{2}+{a_{22}}^{2}}}$$

$$\text{shared heritability}=\frac{\left|a_{11}a_{12}\right|}{\left(\left|a_{11}a_{12}\right|+\left|c_{11}c_{12}\right|+\left|e_{11}e_{12}\right|\right)}$$

For shared heritability, absolute values of the path coefficients were used to calculate the proportion of genetic contribution against the total contribution, which was calculated as the sum of each factor's contribution (Coan, 2003).

For both univariate and bivariate heritability analysis, full ACE models were compared to CE models, which did not include genetic factors, to measure genetic influence. Furthermore, the full ACE models were also compared with AE and E models and corresponding Akaike Information Criteria were calculated.

All statistical analyses including heritability analyses were performed using R statistical software (version 3.6.2, Vienna, Austria). All twin heritability analyses were performed using OpenMx (version 2.12.2) and umx (version 2.9.9) packages within R, which allowed structural equation modeling to estimate heritability (Neale et al., 2016). Covariates were adjusted using a residual approach in R with standardized values, and then heritability was assessed for each type of adjustment in both univariable and bivariate analyses.

## 3 RESULTS

### 3.1 Reliability and validity of the automated dPVS segmentation method

Automatically calculated volumes of BGdPVS and WMdPVS showed excellent reliability between the test and retest datasets (ICC of BGdPVS = .983, ICC of WMdPVS = .988; both *p* < .001; Table S1).

In the 10 randomly selected subjects, the automated dPVS segmentation method also showed strong consistency with manual segmentation results, showing an ICC of .978 for BGdPVS and .993 for WMdPVS volumes (Table S2). The automated dPVS segmentation method yielded 0% and 8.1 ± 10% for false‐positive and false‐negative fraction volumes, respectively, with reference to the manual segmentation as the gold standard.

### 3.2 Characteristics of the study cohort

The baseline characteristics of all subjects are summarized in Table 1. Group‐wise comparisons of the clinical and volumetric variables among the three groups are summarized in Table S3. Both MZ and DZ twin pairs were older (mean age, 29 years old vs. 27 years old, *p* < .001 and *p* = .003, respectively) and consisted of fewer male subjects (41.3 and 38.0% vs. 53.8%, *p* = .033 and *p* = .018, respectively) than nontwin siblings. The most predominant ethnic group was White (*n* = 530), followed by Black (*n* = 88), and Asian/Natural Hawaiian (*n* = 40). Twenty‐six subjects answered that they belonged to more than one ethnic group, while 16 subjects did not know or report their ethnic group. Excluding these 42 subjects whose ethnicities were not specified, the frequency of ethnicities was not significantly different between groups. BG volumes were significantly greater in nontwin siblings than in MZ twins (23.3 vs. 22.3 cm^3^, *p* = .048). Every participant had dPVS in both the BG (range, 0.092–0.400 cm^3^) and WM (range, 0.503–5.933 cm^3^). BGdPVS volumes were greater in DZ twins than in nontwin siblings (0.240 cm^3^ vs. 0.225 cm^3^, *p* = .002). No significant differences were found in other characteristics.

**TABLE 1:** Clinical and volumetric variables

	MZ twins	DZ twins	Nontwin siblings
	(*N* = 276)	(*N* = 158)	(*N* = 266)
Age	29.0 [27.0;32.0]	29.0 [27.0;32.0]	27.0 [24.0;31.0]
Sex, male	114 (41.3%)	60 (38.0%)	143 (53.8%)
Ethnicity			
White	234 (84.8%)	132 (83.5%)	164 (61.7%)
Black or African American	24 (8.7%)	20 (12.7%)	44 (16.5%)
Asian/natural Hawaiian/other Pacific islander	10 (3.6%)	4 (2.5%)	26 (9.8%)
More than one	4 (1.4%)	2 (1.3%)	20 (7.5%)
Unknown or not reported	4 (1.4%)	0 (0%)	12 (4.5%)
Systolic BP	122.0 [113.0;132.0]	121.5 [114.0;131.0]	121.5 [113.0;132.0]
Diastolic BP	76.5 [69.0;82.0]	76.0 [68.0;82.0]	76.0 [69.0;83.0]
Pulse pressure	46.0 [40.0;53.0]	46.0 [40.0;54.0]	46.0 [40.0;52.0]
HbA1c (%)	5.1 [0.0; 5.3]	5.0 [0.0; 5.3]	5.1 [0.0; 5.4]
PSQI	4.0 [3.0; 6.0]	4.0 [2.0; 6.0]	4.0 [3.0; 7.0]
MMSE	29.0 [29.0;30.0]	29.0 [28.0;30.0]	29.0 [29.0;30.0]
BG (cm^3^)	22.3 [21.1; 24.1]	22.7 [21.2; 24.7]	23.3 [21.7; 24.7]
WM (cm^3^)	434.4 [405.1; 474.1]	437.6 [408.5; 480.1]	448.9 [404; 485.3]
ICV (cm^3^)	1,589.2 [1,459.8; 1,695.2]	1,554.9 [1,471.6; 1718.3]	1,607.5 [1,486.4; 1742]
BGdPVS (cm^3^)	0.229 [0.198; 0.285]	0.240 [0.207; 0.294]	0.225 [0.184; 0.265]
WMdPVS (cm^3^)	1.951 [1.448; 2.717]	1.918 [1.405; 2.431]	1.744 [1.314; 2.40]

*Note:* Data are expressed as medians with interquartile ranges or as numbers with percentages in parentheses.

Abbreviations: BG, basal ganglia; BGdPVS, dilated perivascular space in basal ganglia; BP, blood pressure; DZ, dizygotic; ICV, intracranial volume; MMSE, mini‐mental state examination; MZ, monozygotic; PSQI, Pittsburg Sleep Quality Index; WM, white matter; WMdPVS, dilated perivascular space in white matter.

### 3.3 Group comparisons of within‐pair differences for subject characteristics

The nontwin siblings had greater differences in age and sex than MZ or DZ twin pairs (all, *p* < .001). The differences in volumes of BGdPVS, WMdPVS, BG, and WM, and ICV were greatest in nontwin siblings followed by DZ and MZ twin pairs in that order (all, *p* < .001). The within‐pair differences in diastolic BP and HbA1c levels were also significantly different between groups (*p* < .001 and *p* = .004, respectively). No significant within‐pair differences were found for the other characteristics (Table 2).

**TABLE 2:** Group comparisons of within‐pair differences in clinical variables

Within‐pair differences	MZ twin pairs	DZ twin pairs	Nontwin sibling pairs		Posthoc analysis^b^		
	(*N* = 138)	(*N* = 79)	(*N* = 133)	*p* value^a^	*p*1	*p*2	*p*3
ΔAge	0.0 [0.0; 0.0]	0.0 [0.0; 0.0]	3.0 [2.0; 5.0]	<.001	NA^d^	<0.001	<0.001
ΔSex	0 (0.0%)	0 (0.0%)	67 (50.4%)	<.001^c^	NA^d^	<0.001^d^	<0.001^d^
ΔSystolic BP	9.0 [4.0; 16.0]	11.0 [5.0; 21.0]	12.0 [6.0; 22.0]	.058			
ΔDiastolic BP	7.0 [3.0; 12.0]	11.0 [6.0;16.0]	8.0 [5.0; 15.0]	.001	<0.001	0.004	0.001
ΔPulse pressure	7.0 [3.0; 14.0]	11.0 [4.0; 15.5]	9.0 [4.0; 15.0]	.306			
ΔHbA1c (%)	0.2 [0.0; 0.7]	0.2 [0.0; 0.8]	0.3 [0.1; 5.2]	.004	<0.001	0.018	0.004
ΔPSQI	2.0 [1.0; 3.0]	2.0 [1.0; 4.0]	2.0 [1.0; 4.0]	.866			
ΔMMSE	1.0 [0.0; 1.0]	1.0 [0.0; 1.0]	1.0 [0.0; 1.0]	.404			
ΔBG (cm^3^)	0.7 [0.3; 1.3]	1.3 [0.5; 2.0]	1.8 [0.9; 3.1]	<.001	<0.001	<0.001	<0.001
ΔWM (cm^3^)	8.0 [4.1; 12.2]	26.4 [12.1; 44.5]	51.0 [28.2; 85.1]	<.001	<0.001	<0.001	<0.001
ΔICV (cm^3^)	31.5 [14.7; 61.5]	85.2 [37.6; 13.4]	160.1 [79.9; 27.2]	<.001	<0.001	<0.001	<0.001
ΔBGdPVS (cm^3^)	0.029 [0.011; 0.053]	0.039 [0.022; 0.091]	0.045 [0.02; 0.075]	<.001	<0.001	<0.001	<0.001
ΔWMdPVS (cm^3^)	0.229 [0.001; 0.394]	0.551 [0.211; 0.985]	0.591 [0.256; 1.242]	<.001	<0.001	<0.001	<0.001

*Note:*Data are expressed as medians with interquartile ranges or as numbers with percentages in parentheses. For variable sex, the number of subjects with opposite sex is shown with its proportions in parentheses. Δ, absolute difference between pairs; p1, MZ twin vs. DZ twin; p2, MZ twin vs. nontwin sibling; p3, DZ twin vs. nontwin sibling.

Abbreviations: BG, basal ganglia; BGdPVS, dilated perivascular space in basal ganglia; BP, blood pressure; DZ, dizygotic; ICV, intracranial volume; MMSE, mini‐mental state examination; MZ, monozygotic; NA, not applicable; PSQI, Pittsburg Sleep Quality Index; WM, white matter; WMdPVS, dilated perivascular space in white matter.

^a^ Kruskal–Wallis Rank Sum Test.

^b^ Tukey's test.

^c^ Chi‐square test.

^d^ Bonferroni correction.

### 3.4 Within‐pair correlations of dPVS volumes

**FIGURE 2:**
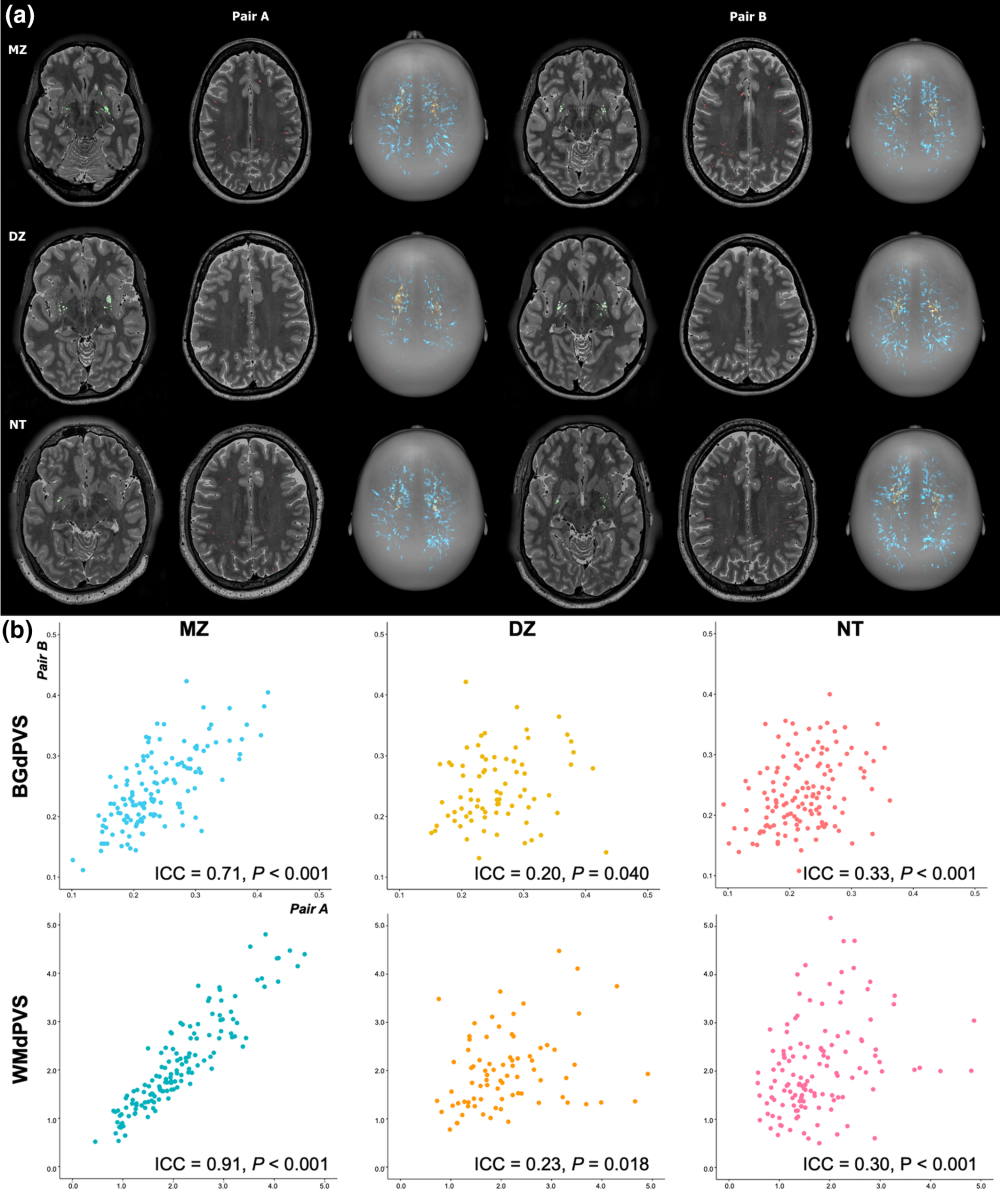
(a) Representative images of MRI‐visible dilated perivascular space in white matter (WMdPVS) (red; blue in three‐dimensional) and dilated perivascular space in basal ganglia (BGdPVS) (green; yellow in three‐dimensional) segmentations of monozygotic (MZ) and dizygotic (DZ) twin pairs and nontwin siblings (NT). Compared to the DZ twins and nontwin siblings, noticeable overlap in dPVS is visible in MZ twins. (b) Scatter plots of BGdPVS (upper row) and WMdPVS (bottom row) volumes between subjects within pair in MZ, DZ twins, and nontwin siblings and corresponding intraclass correlation coefficients

Representative images and correlation plots of BGdPVS and WMdPVS volumes between twin and nontwin siblings are shown in Figure 2. MZ twin pairs showed higher correlations in both BGdPVS and WMdPVS (ICC = .71 and ICC = .91, both *p* < .001), than DZ twin pairs (ICC = .20, *p* = .040 and ICC = .23, *p* = .018) and nontwin siblings (ICC = .33 and ICC = .30, both *p* < .001).

### 3.5 Effect of clinical and volumetric variables on dPVS volumes

Older age and male sex were significantly associated with larger dPVS volume (both, *p* < .001). The effect of sex was no longer significant after adjusting for ICV (*p* = .503 for BGdPVS; *p* = .143 for WMdPVS), while the effect of age remained significant after adjusting for ICV or all other variables (*P*s < .001). The White ethnic group adjusted for age and sex was associated with larger BGdPVS than the nonWhite group (*p* = .004). However, after adding ICV as a covariate, the significance disappeared (*p* = .114). All volumetric variables adjusted for age and sex also significantly affected dPVS volume (all, *p* < .01). However, none of the BP variables adjusted for age and sex were associated with dPVS volume (Tables S4 and S5). Therefore, in subsequent heritability analyses we adjusted for age, sex, regional volumes, ICV, and ethnic group, all of which were significantly associated with dPVS volume. Besides, we also included pulse pressure as a covariate in heritability analyses as hypertension and arterial pulsation are well‐known key factors in the development of dPVS.

### 3.6 Univariate heritability analysis

The results of the univariate heritability analyses are shown in Table 3. Overall, the results of the univariate analyses suggest higher heritability in WMdPVS volume (87.4–90.2%) than in BGdPVS volume (61.1–65.8%). Adjusting for regional volume, ICV, and ethnic group resulted in slightly lower heritability estimates, while adjusting for pulse pressure resulted in minimal changes in heritability estimates (Tables S6 and S7). The heritability estimates of the White ethnic group alone also resulted in slightly lower estimates but overall were within an acceptable range (Table S8). The standardized path coefficients of genetic [A], shared environment [C], and unique environment [E] factors for dPVS volumes in each model were plotted in Figure S1. In all analyses, heritability measures were significant when the full ACE models were compared to the model without the genetic factor (CE) (*p* < 0.001; Table S9).

**TABLE 3:** Univariate heritability estimates (*h* ^2^) with 95% confidence intervals for BGdPVS and WMdPVS volumes

	Unadjusted (age, sex)	Partially adjusted (1) (age, sex, regional volume^a^)	Partially adjusted (2) (age, sex, ICV)	Completely adjusted (age, sex, regional volume^a^, ICV)
BGdPVS	0.658 [0.487, 0.733]	0.650 [0.453, 0.725]	0.611 [0.418, 0.694]	0.611 [0.418, 0.694]
WMdPVS	0.902 [0.827, 0.925]	0.874 [0.795, 0.904]	0.884 [0.802, 0.911]	0.875 [0.795, 0.904]

*Note:* All *h* ^*2*^ showed *p* < .001 when the full model (ACE) was compared to the corresponding model that did not include the genetic factors (CE).

Abbreviations: BGdPVS, dilated perivascular space in basal ganglia; ICV, intracranial volume; WMdPVS, dilated perivascular space in white matter.

^a^ Volumes of WM for WMdPVS and BG for BGdPVS.

### 3.7 Bivariate heritability analysis

The results of the bivariate heritability analyses are shown in Table 4. As in univariate analysis, heritability measures were significant when the full ACE models were compared with the CE model without the genetic factor (*p* < .001; Table S10). Genetic correlations (*r*_g_) were low to moderate in BGdPVS and WMdPVS volumes with surrounding regional volumes (0.37 and 0.43, respectively) and ICV (0.39 and 0.37, respectively), and between each other (0.30). Pulse pressure had low genetic correlation with both BGdPVS and WMdPVS volumes (0.14 and 0.29, respectively). In spite of low to moderate genetic correlation, both BGdPVS and WMdPVS volumes had high shared heritability estimates with corresponding regional volumes (71.8 and 97.2%, respectively), ICV (95.2 and 99.9%, respectively), and other regional dPVS volumes (82.8%). The shared heritability estimate of pulse pressure with both BGdPVS and WMdPVS volumes was 55.6 and 56.5%, respectively; however, these results might not be reliable due to the wide range of the 95% confidence intervals. The standardized path coefficients of genetics [A], shared environment [C], and unique environment [E] between dPVS volumes and BG volume, WM volume, ICV, and pulse pressure were plotted in Figure S2.

**TABLE 4:** Bivariate shared heritability estimates (*shared h* ^*2*^) and genetic correlations (*r*_g_) between dPVS and regional volume, intracranial volume, pulse pressure, and different regional dPVS

	*r*_g_	*Shared h* ^*2*^
BGdPVS		
BG	.37	0.718 [0.580, 0.999]
ICV	.39	0.952 [0.744, 0.999]
Pulse pressure	.14	0.556 [0.000, 0.999]
WMdPVS	.30	0.828 [0.597, 0.932]
WMdPVS		
WM	.43	0.972 [0.837, 0.999]
ICV	.37	0.999 [0.823, 0.999]
Pulse pressure	.29	0.565 [0.000, 0.999]
BGdPVS	.30	0.828 [0.597, 0.932]

*Note:* All were statistically significant (*p* < .001) when the ACE model was compared to the CE model.

Abbreviations: BG, basal ganglia; BGdPVS, dilated perivascular space in basal ganglia; dPVS, dilated perivascular space; ICV, intracranial volume; WM, white matter; WMdPVS, dilated perivascular space in white matter.

## 4 DISCUSSION

The current study assessed the heritability of dPVS volume in healthy young twins and nontwin siblings in a well‐controlled large dataset with high quality images. There were three main findings in this study. First, our results support a previous study's finding by also showing highly heritable dPVS burden (Duperron et al., 2018). In the current study, the heritability estimates of both BGdPVS and WMdPVS volumes were high, and even higher than those reported in the elderly cohort (Duperron et al., 2018). Second, the heritability estimates were higher in WMdPVS volume than in BGdPVS volume, supporting differential genetic influences on dPVS by location. Lastly, among the well‐known risk factors for dPVS, age was significantly associated with dPVS volume even in young subjects, but the effect of BP on dPVS volume was limited in its normal range.

dPVS burden has usually been assessed with subjective visual rating scales in most studies (Duperron et al., 2018; Laveskog, Wang, Bronge, Wahlund, & Qiu, 2018). Visual assessment is easily performed in clinical settings, but is time‐consuming, rater dependent, and prone to ceiling and floor effects, and these inherent limitations prevent the precise assessment of dPVS burden and its heritability. Therefore, we developed a fully automated segmentation algorithm for the quantitative and objective evaluation of dPVS burden. Although, the dPVS volumes measured by our automated method were slightly smaller than manually segmented dPVS volumes in some subjects, the automated method revealed results strongly consistent with those of manual assessment and without false‐positive findings. Moreover, the automated method showed excellent reliability in 45 subjects who repeated MRI scanning within relatively short intervals. Therefore, this study demonstrated that the automated segmentation algorithm could accurately and reliably assess dPVS burden in the HCP dataset.

When comparing within‐pair differences, differences in dPVS volume between the MZ twin pairs were the smallest, followed by those between the DZ twin pairs and nontwin siblings, which suggests genetic effects on dPVS volume. In line with this result, the heritability estimates were high in both BGdPVS and WMdPVS in our healthy young cohort. The heritability estimates were higher than those reported in a previous study on elderly subjects that had a high frequency of vascular risk factors [BGdPVS (current: 61.0–65.8% vs. previous 40%); WMdPVS (current: 87.4–90.3% vs. previous: 79%)] (Duperron et al., 2018). This difference might be due to the relatively brief exposure of healthy young adults to environmental impacts, allowing genetic contribution to remain significant in the younger population. However, as genome‐wide genotyping which was used in a previous study (Duperron et al., 2018) reveals lower heritability estimates compared to twin studies (Visscher & Goddard, 2015), findings of these studies and ours need to be compared and interpreted with caution. Future studies with the same heritability analytic methods may be able to draw more convincing conclusions on this issue.

Similar to the previous study (Duperron et al., 2018), the heritability of the dPVS volume was higher in WM than in BG in our study, suggesting a distinct genetic influence on dPVS according to location. Growing evidence indicates different risk factors and clinical manifestations for BGdPVS and WMdPVS (Andreas Charidimou et al., 2017; Duperron et al., 2018; Martinez‐Ramirez et al., 2013; Roher et al., 2003). BGdPVS burden has been associated with hypertension, hypertensive angiopathy, and vascular cognitive impairment (Chen et al., 2019; Hansen, Cain, Thomas, & Jackson, 2015), while WMdPVS burden has been associated with cerebral amyloid angiopathy and Alzheimer's disease (Chen et al., 2019; van Veluw et al., 2016). In Alzheimer's disease and cerebral amyloid angiopathy, amyloid β deposition mainly occurs in the arteries of the cerebral cortex and leptomeninges (Vinters & Gilbert, 1983), potentially increasing arterial stiffness and compromising normal arterial pulsation in these areas, while brain regions commonly affected by hypertensive arteriopathy (i.e., BG, brain stem and cerebellum) (Andreas Charidimou et al., 2017) are usually free from amyloid β deposition (Mandybur, 1975). As the arterial pulsation is a key driver of fluid movement through PVS (Mestre et al., 2018), arterial amyloid β deposition could be a possible explanation for the fluid stagnation and PVS dilatation in WM near the cortex. Likewise, the arterial stiffness in BG caused by hypertension, might, at least in part, cause BGdPVS. Given that Alzheimer's disease shows higher heritability (60–80%) than hypertension (30–60%; Gatz et al., 2006; Kupper et al., 2005; Scherbakov, Von Haehling, Anker, Dirnagl, & Doehner, 2013; Shih & O'Connor, 2008), the distinct heritability of associated diseases might be related to the higher heritability of WMdPVS than BGdPVS. Furthermore, although there have been conflicting data, a previous study even found a direct association between WMdPVS and the apolipoprotein E ε4 allele (Roher et al., 2003). Meanwhile, the lower heritability of BGdPVS might also be due to its higher susceptibility to environmental factors. Although the underlying process is still unknown, the anatomical differences of PVS according to location might be responsible for its distinct vulnerability to the environment. BGdPVS has two layers while WMdPVS has one for the pial membrane and surrounds more proximal arterioles while WMdPVS has smaller arterioles or capillaries (Pollock, Hutchings, Weller, & Zhang, 1997; Zhang, Inman, & Weller, 1990). As proximal arterioles have looser endothelial tight junctions than smaller arterioles or capillaries (Bechmann, Galea, & Perry, 2007), they may be affected more by endothelial damage induced by hypertension or other environmental insults (Wardlaw, Smith, & Dichgans, 2013). Moreover, BGdPVS directly communicates with the adjacent basal subarachnoid space (Bouvy et al., 2014) and lenticulostriatal arteries are exposed to higher BP than cortical arteries (Yamaguchi, Fukuyama, Yamauchi, & Kimura, 1991), which might also make BGdPVS more vulnerable to hypertension or toxic materials circulating in the cerebrospinal fluid. However, there is not enough conclusive evidence and longitudinal studies must be performed to define a distinct pathogenesis of dPVS according to location.

Age, male sex, and hypertension are well‐known risk factors for dPVS, especially for BGdPVS (Martinez‐Ramirez et al., 2013; Y.‐C. Zhu et al., 2010). Our results also revealed the significant effect of age and sex on both BGdPVS and WMdPVS volumes. However, after adjusting for ICV, the significance of sex no longer remained, while the effect of age remained significant even after all other clinical and volumetric variables were included as covariates. This finding suggests that the age effect may kick in early even at a very young age; while the sex effect might be, at least in part, due to the larger brain volumes of the male sex. The association between high BP and dPVS burden has been consistently reported (Y. C. Zhu et al., 2010), and a previous study suggested that the pulsatile component might have a more important role in the pathogenesis of PVS dilatation (Del Brutto & Mera, 2018). However, our findings showed that pulse pressure was not significantly correlated with dPVS volume. Furthermore, the effect of pulse pressure on dPVS heritability was weak. Considering that our cohort was made up of healthy individuals with normal‐ranged BP, this finding suggests a limited phenotypic interaction between BP and dPVS in the normal ranges of BP. Therefore, we assume that BP might only affect PVS dilatation with significance when it is above a certain value.

The effect of ethnic group on dPVS has rarely been studied. A previous large population‐based study reported a higher prevalence of BGdPVS and WMdPVS in White patients than Chinese patients who experienced a transient ischemic attack or ischemic stroke (Lau et al., 2017). In line with this result, our study also found larger BGdPVS volume in the White ethnic group after adjusting for age and sex. Ethnic differences in brain size (Hsu et al., 2018; Rushton & Ankney, 1996) might affect the results. In line with our assumption, the significance of ethnicity did not remain after adding ICV as a covariate in our study. However, this result should be interpreted with caution as the number of subjects was imbalanced between ethnic groups, and further studies are warranted for validation.

There are severe limitations that need to be addressed in this study. First, this is a retrospective study with a publicly available cohort, and as such, longitudinal follow‐up was not possible, which limits our understanding of the clinical implications of dPVS at this time. For example, we cannot elucidate whether PVS dilatation found in healthy young adults was a risk factor for future small vessel disease or cognitive decline; it could simply be a benign inherited trait. Similarly, we cannot know what clinical relevance our findings have for different cohorts such as older individuals with cerebral small vessel disease or neurodegenerative pathology at this time. Second, the reliability of automated dPVS segmentation was assessed with MRI scans obtained at different time points. Although the time interval was short—as we demonstrated that age affected dPVS dilatation even in young adults—the dPVS volumes might differ between the two MRI scans. In our study, dPVS volumes on the second scan were larger than the first scan (mean volume increases were 0.013 cm^3^ for BGdPVS and 0.708 cm^3^ for WMdPVS, data not shown). We believe that this difference might have lowered the reliability of the automated segmentation method, and eliminating this confounding factor by having MRI scans performed on the same day would further confirm the reliability of our algorithm. Third, the automatic segmentation algorithm was built based on T2‐weighted images only. Although our algorithm did not degrade its function in our young healthy population, a future study employing multiple sequences including FLAIR images for the automatic segmentation algorithm might help differentiate dPVS mimicking WM hyperintensities or old lacunes, and therefore, improve the dPVS segmentation accuracy, particularly in the elderly population. Fourth, heritability is a ratio, namely, a relative measure. It is affected by variations in cohort and does not directly reflect genetic determination. Lastly, we had an issue with negative path coefficients on bivariate heritability analysis. Although we adopted a previously reported method to calculate shared heritability using absolute values (Coan, 2003), there is not enough research on how to handle and interpret these negative values in heritability analysis (Steinsaltz, Dahl, & Wachter, 2017). As seen in our study, a few reports have also demonstrated negative path coefficients (Kendler, Myers, Dick, & Prescott, 2010; Tanguay‐Garneau et al., 2020). Moreover, a recent study has suggested that while these negative values might be random noise or results of model misspecification, they might also reflect a real physical feature of the biological process (Steinsaltz et al., 2017). We hope our study can motivate future research to unveil the significance of these negative values. Despite these limitations, we believe that our results might support the substantial genetic contribution of PVS dilatation.

In summary, the present study provides evidence for a substantial genetic contribution to dPVS burden in young healthy adults. Older age was associated with higher dPVS burden even in young adults, while BP had limited association with dPVS in its normal range. Future longitudinal studies are needed to explore the clinical implications of dPVS in healthy young adults and understand the relevant environmental risk factors of dPVS.

## CONFLICT OF INTEREST

The authors declare no competing interests.

## Supporting information


**Appendix** **S1:** Supplementary Information

## Data Availability

The data of the Human Connectome Project is publicly available (http://www.humanconnectomeproject.org/). The specific codes used for automated segmentation can be shared upon reasonable request by a qualified investigator.
