# Supplementary material for: MRI‐visible dilated perivascular spaces in healthy young adults: A twin heritability study
Source: Hum Brain Mapp. 2020 Sep 8;41(18):5313–24. doi: 10.1002/hbm.25194 (PMC7670636; doi:10.1002/hbm.25194)
Supplement: Supplementary file 1 — Appendix S1: Supplementary Information [file HBM-41-5313-s001.docx]

**Supporting Table S1.** Intraclass correlation coefficients of the automatically calculated volumes of BGdPVS and WMdPVS between test and retest datasets (n=45)

| BGdPVS (cm^3^) | | WMdPVS (cm^3^) | |
| --- | --- | --- | --- |
| test | retest | test | retest |
| 0.295 | 0.289 | 1.978 | 2.205 |
| 0.274 | 0.256 | 1.639 | 1.479 |
| 0.259 | 0.249 | 1.517 | 1.544 |
| 0.210 | 0.199 | 1.917 | 1.646 |
| 0.171 | 0.179 | 1.864 | 1.705 |
| 0.242 | 0.247 | 2.125 | 1.690 |
| 0.130 | 0.154 | 1.792 | 1.809 |
| 0.216 | 0.212 | 1.731 | 1.436 |
| 0.209 | 0.184 | 1.687 | 1.527 |
| 0.267 | 0.265 | 0.463 | 0.540 |
| 0.145 | 0.153 | 2.384 | 2.280 |
| 0.205 | 0.214 | 1.752 | 2.079 |
| 0.285 | 0.261 | 1.140 | 0.973 |
| 0.317 | 0.340 | 2.033 | 2.143 |
| 0.209 | 0.192 | 1.945 | 1.944 |
| 0.172 | 0.181 | 4.112 | 4.100 |
| 0.208 | 0.162 | 1.385 | 1.041 |
| 0.256 | 0.246 | 1.081 | 0.995 |
| 0.264 | 0.236 | 1.599 | 1.409 |
| 0.183 | 0.190 | 1.580 | 1.561 |
| 0.230 | 0.250 | 1.726 | 1.696 |
| 0.259 | 0.260 | 0.863 | 0.852 |
| 0.162 | 0.182 | 2.309 | 2.449 |
| 0.210 | 0.215 | 3.360 | 3.199 |
| 0.173 | 0.177 | 1.672 | 1.653 |
| 0.228 | 0.241 | 1.456 | 1.465 |
| 0.198 | 0.213 | 1.426 | 1.217 |
| 0.170 | 0.191 | 2.827 | 3.300 |
| 0.407 | 0.405 | 3.433 | 3.364 |
| 0.251 | 0.226 | 1.197 | 1.065 |
| 0.187 | 0.166 | 2.109 | 2.271 |
| 0.343 | 0.332 | 1.915 | 1.938 |
| 0.203 | 0.189 | 1.997 | 2.097 |
| 0.155 | 0.162 | 4.332 | 4.156 |
| 0.275 | 0.286 | 1.553 | 1.566 |
| 0.155 | 0.145 | 2.147 | 2.322 |
| 0.164 | 0.184 | 2.876 | 2.729 |
| 0.340 | 0.323 | 1.421 | 1.332 |
| 0.243 | 0.236 | 2.192 | 2.095 |
| 0.123 | 0.134 | 2.372 | 2.514 |
| 0.276 | 0.295 | 2.016 | 2.081 |
| 0.223 | 0.216 | 0.555 | 0.560 |
| 0.422 | 0.385 | 2.486 | 2.471 |
| 0.235 | 0.235 | 2.306 | 2.318 |
| 0.178 | 0.172 | 1.912 | 1.783 |
| ICC [95% CI] | *P* value | ICC [95% CI] | *P* value |
| 0.983 [0.968, 0.990] | <0.001 | 0.988 [0.979, 0.994] | <0.001 |

BGdPVS = dilated perivascular space in basal ganglia, CI=confidence interval, ICC=intraclass correlation coefficient, WMdPVS = dilated perivascular space in white matter.

**Supporting Table S2.** Reliability assessment of dPVS volume measured on one slice in randomly selected subjects (n=10)

| BGdPVS^†^ (cm^3^) | | WMdPVS^‡^ (cm^3^) | |
| --- | --- | --- | --- |
| Manual | Automated | Manual | Automated |
| 0.0058 | 0.0041 | 0.0209 | 0.0185 |
| 0.0051 | 0.0051 | 0.0573 | 0.0545 |
| 0.0161 | 0.0158 | 0.0405 | 0.0374 |
| 0.0038 | 0.0024 | 0.0158 | 0.0158 |
| 0.0165 | 0.0141 | 0.0213 | 0.0213 |
| 0.0106 | 0.0086 | 0.0178 | 0.0178 |
| 0.0261 | 0.0230 | 0.0466 | 0.0466 |
| 0.0075 | 0.0058 | 0.0350 | 0.0309 |
| 0.0192 | 0.0168 | 0.0549 | 0.0549 |
| 0.0141 | 0.0110 | 0.0552 | 0.0490 |
| ICC [95% CI] | *P* value | ICC [95% CI] | *P* value |
| 0.978  [0.261, 0.996] | 0.012 | 0.993  [0.943, 0.998] | <0.001 |

^†^BGdPVS volume measured at the level of anterior commissure

^‡^WMdPVS volume measured at the upper end of corpus callosum

BGdPVS = dilated perivascular space in basal ganglia, CI=confidence interval, ICC=intraclass correlation coefficient, WMdPVS = dilated perivascular space in white matter.

**Supporting Table S3.** Group-wise comparison of clinical and volumetric variables^†^

|  | MZ vs. NT | | | DZ vs. NT | | | MZ vs. DZ | | |
| --- | --- | --- | --- | --- | --- | --- | --- | --- | --- |
|  | Estimate | SE | *P* value* | Estimate | SE | *P* value* | Estimate | SE | *P* value* |
| Age | 1.657 | 0.400 | <0.001 | 1.526 | 0.467 | 0.003 | 0.132 | 0.464 | 1.000 |
| Sex, male^†^ | -1.305 | 0.512 | 0.033 | -1.684 | 0.615 | 0.018 | 0.379 | 0.593 | 1.000 |
| Ethnic group, non-White^†‡^ | -1.164 | 1.772 | 1.000 | 0.927 | 2.004 | 1.000 | -0.237 | 2.245 | 1.000 |
| Systolic BP | 2.771 | 1.950 | 0.468 | 1.665 | 2.279 | 1.000 | 1.106 | 2.264 | 1.000 |
| Diastolic BP | 1.758 | 1.349 | 0.579 | 0.034 | 1.577 | 1.000 | 1.724 | 1.566 | 0.816 |
| Pulse BP | 1.014 | 1.057 | 1.000 | 1.632 | 1.234 | 0.561 | -0.618 | 1.227 | 1.000 |
| HbA1c (%) | 0.078 | 0.235 | 1.000 | -0.322 | 0.274 | 0.726 | 0.399 | 0.273 | 0.432 |
| PSQI | -0.537 | 0.247 | 0.090 | -0.452 | 0.288 | 0.354 | -0.085 | 0.286 | 1.000 |
| MMSE | 0.031 | 0.091 | 1.000 | -0.022 | 0.106 | 1.000 | 0.053 | 0.105 | 1.000 |
| BG (cm^3^) | -0.645 | 0.268 | 0.048 | -0.123 | 0.313 | 1.000 | -0.523 | 0.311 | 0.279 |
| WM (cm^3^) | -9.658 | 5.848 | 0.300 | -0.703 | 6.836 | 1.000 | -8.955 | 6.789 | 0.564 |
| ICV (cm3) | -39.810 | 20.040 | 0.144 | -22.040 | 23.420 | 1.000 | -17.773 | 23.263 | 1.000 |
| BGdPVS (cm^3^) | 0.012 | 0.006 | 0.147 | 0.025 | 0.007 | 0.002 | -0.013 | 0.007 | 0.255 |
| WMdPVS (cm^3^) | 0.181 | 0.097 | 0.195 | 0.102 | 0.114 | 1.000 | 0.079 | 0.113 | 1.000 |

^†^Generalized linear mixed model was used; for other continuous variables, linear mixed models were used including subject group as a fixed effect and twin pair as a random effect. The group written after “vs.” is the reference group.

^‡^Only subjects belonging to the White and non-White (i.e., Black or African American, Asian, natural Hawaiian, or other Pacific Islander) ethnic groups (n=658) were included and the White group was used as the reference group.

* *P* values for testing pair-wise group differences in coefficients corrected for multiple comparisons with the Bonferroni method.

BG = basal ganglia, BGdPVS = dilated perivascular space in basal ganglia, BP = blood pressure, DZ = dizygotic twins, ICV = intracranial volume, MMSE = mini-mental state examination, MZ = monozygotic twins, NT = non-twin siblings, PSQI = Pittsburg Sleep Quality Index, SE = standard error, WM = white matter, WMdPVS = dilated perivascular space in white matter.

**Supporting Table S4.** Effect of clinical and volumetric variables on the BGdPVS volume (mm^3^)

|  | Estimate | SE | df | *t* value | *P* value |
| --- | --- | --- | --- | --- | --- |
| Age^†^ | 4.396 | 0.667 | 593.123 | 6.593 | <0.001 |
| Sex, male^†^ | 15.886 | 4.858 | 671.657 | 3.270 | 0.001 |
| Ethnic group, non-White^‡^ | -19.913 | 6.868 | 328.151 | -2.899 | 0.004 |
| Systolic BP | 0.036 | 0.107 | 666.380 | 0.334 | 0.738 |
| Diastolic BP | 0.076 | 0.156 | 675.485 | 0.483 | 0.629 |
| Pulse BP | 0.001 | 0.183 | 632.100 | 0.006 | 0.995 |
| HbA1c (%) | 1.624 | 0.892 | 678.900 | 1.820 | 0.069 |
| PSQI | 0.281 | 0.747 | 621.838 | 0.376 | 0.707 |
| MMSE | 0.899 | 2.122 | 642.609 | 0.424 | 0.672 |
| BG (cm^3^) | 4.213 | 1.096 | 635.286 | 3.844 | <0.001 |
| WM (cm^3^) | 0.443 | 0.048 | 648.383 | 9.134 | <0.001 |
| ICV (cm^3^) | 0.097 | 0.016 | 646.643 | 6.192 | <0.001 |

Linear mixed-effects model analyses were performed including each variable as a fixed effect and twin pair as a random effect.

^†^Baseline model fit was adopted for age and sex; estimates for all other clinical variables were calculated by adding age and sex as covariates to the baseline model. The effect of sex was no longer significant after adjusting for ICV (*P* = 0.503), while the effect of age remained significant after adjusting for ICV only or all other variables (*Ps* < 0.001). The effect of ethnic group was also not significant after adding ICV as a covariate (*P* = 0.114).

^‡^Only subjects belonging to the White and non-White (i.e., Black or African American, Asian, natural Hawaiian, or other Pacific Islander) ethnic groups were included and the White group was used as the reference group.

BG = basal ganglia, BGdPVS = dilated perivascular space in basal ganglia, BP = blood pressure, df = degree of freedom, ICV = intracranial volume, MMSE = mini-mental state examination, PSQI = Pittsburg Sleep Quality Index, SE = standard error, WM = white matter, WMdPVS = dilated perivascular space in white matter.

**Supporting Table S5.** Effect of clinical and volumetric variables on the WMdPVS volume (mm^3^)

|  | Estimate | SE | df | t value | *P* value |
| --- | --- | --- | --- | --- | --- |
| Age^†^ | 44.450 | 10.220 | 643.010 | 4.349 | <0.001 |
| Sex, male^†^ | 503.420 | 69.910 | 692.590 | 7.201 | <0.001 |
| Ethnic group, non-White^‡^ | 8.298 | 107.808 | 328.178 | 0.077 | 0.939 |
| Systolic BP | 1.619 | 1.506 | 608.638 | 1.075 | 0.283 |
| Diastolic BP | 2.049 | 2.207 | 621.222 | 0.929 | 0.353 |
| Pulse BP | 1.909 | 2.556 | 571.609 | 0.747 | 0.456 |
| HbA1c (%) | -3.636 | 12.674 | 629.315 | -0.287 | 0.774 |
| PSQI | 17.836 | 10.404 | 560.159 | 1.714 | 0.087 |
| MMSE | -19.625 | 29.739 | 582.937 | -0.66 | 0.510 |
| BG (cm^3^) | 45.774 | 16.243 | 679.991 | 2.818 | 0.005 |
| WM (cm^3^) | 7.049 | 0.706 | 689.618 | 9.982 | <0.001 |
| ICV (cm^3^) | 1.725 | 0.227 | 690.425 | 7.598 | <0.001 |

Linear mixed-effects model analyses were performed including each variable and subject group as fixed effects and individuals within pair as random effects.

^†^Baseline model fit was adopted for age and sex; estimates for all other clinical variables were calculated by adding age and sex as covariates to the baseline model. The effect of sex was not significant after adjusting for ICV (*P* = 0.143), while the effect of age remained significant after adjusting for ICV only or all other variables (*Ps* < 0.001). The effect of ethnic group still remained nonsignificant after adding ICV.

^‡^Only subjects belonging to the White and non-White (i.e., Black or African American, Asian, natural Hawaiian, or other Pacific Islander) ethnic groups were included and the White group was used as the reference group.

BG = basal ganglia, BGdPVS = dilated perivascular space in basal ganglia, BP = blood pressure, df = degree of freedom, ICV = intracranial volume, MMSE = mini-mental state examination, PSQI = the Pittsburg Sleep Quality Index, SE = standard error, WM = white matter, WMdPVS = dilated perivascular space in white matter.

**Supporting Table S6.** Univariate heritability estimates (*h^2^*) with pulse pressure as an additional covariate

|  | Unadjusted  (age, sex, pulse pressure) | Partially adjusted (1)  (age, sex, regional volume^†^, pulse pressure) | Partially adjusted (2)  (age, sex, ICV, pulse pressure) | Completely adjusted (age, sex, regional volume^*^, ICV, pulse pressure) |
| --- | --- | --- | --- | --- |
|  | *h^2^* [95% CI] | | | |
| BGdPVS | 0.657 [0.485, 0.732] | 0.650 [0.453, 0.725] | 0.611 [0.417, 0.694] | 0.610 [0.418, 0.694] |
| WMdPVS | 0.901 [0.824, 0.924] | 0.874 [0.794, 0.903] | 0.883 [0.730, 0.910] | 0.874 [0.794, 0.904] |

All *h^2^* showed *P* < 0.001 when the full model (ACE) was compared to the corresponding model without the genetic effect (CE).

^†^Volumes of WM for WMdPVS and BG for BGdPVS.

CI=confidence interval, ICV=intracranial volume.

**Supporting Table S7.** Univariate heritability estimates (*h^2^*) with ethnic group as an additional covariate

|  | Unadjusted  (age, sex, ethnic group) | Partially adjusted (1)  (age, sex, regional volume^†^, ethnic group) | Partially adjusted (2)  (age, sex, ICV, ethnic group) | Completely adjusted  (age, sex, regional volume^†^, ICV, ethnic group) |
| --- | --- | --- | --- | --- |
|  | *h^2^* [95% CI] | | | |
| BGdPVS | 0.640 [0.480, 0.722] | 0.634 [0.455, 0.715] | 0.599 [0.411, 0.687] | 0.598 [0.412, 0.687] |
| WMdPVS | 0.899 [0.818, 0.923] | 0.863 [0.773, 0.896] | 0.873 [0.782, 0.903] | 0.864 [0.773, 0.896] |

Only 658 subjects of the White and non-White (i.e., Black or African American, Asian, natural Hawaiian, or other Pacific Islander) ethnic groups were included.

All *h^2^* showed *P* < 0.001 when the full model (ACE) was compared to the corresponding model without the genetic effect (CE).

†Volumes of WM for WMdPVS and BG for BGdPVS.

CI=confidence interval, ICV=intracranial volume.

**Supporting Table S8.** Univariate heritability estimates (*h^2^*) in White ethnic group

|  | Unadjusted  (age, sex) | Partially adjusted (1) (age, sex, regional volume^†^) | Partially adjusted (2)  (age, sex, ICV) | Completely adjusted  (age, sex, regional volume^†^, ICV) |
| --- | --- | --- | --- | --- |
|  | *h^2^* [95% CI] | | | |
| BGdPVS | 0.646 [0.480, 0.734] | 0.638 [0.455, 0.726] | 0.602 [0.407, 0.698] | 0.600 [0.409, 0.696] |
| WMdPVS | 0.906 [0.802, 0.930] | 0.858 [0.752, 0.894] | 0.871 [0.759, 0.904] | 0.858 [0.752, 0.894] |

Only 530 subjects of the White ethnic groups were included.

All *h^2^* showed *P* < 0.001 when the full model (ACE) was compared to the corresponding model without the genetic effect (CE).

†Volumes of WM for WMdPVS and BG for BGdPVS.

CI=confidence interval, ICV=intracranial volume.

**Supporting Table S9.** Comparison of ACE with AE, CE, and E models in univariate heritability analysis

|  | Model | Unadjusted^†^ | | | | Partially adjusted (1)^‡^ | | | | Partially adjusted (2)^§^ | | | | Completely adjusted^¶^ | | | |
| --- | --- | --- | --- | --- | --- | --- | --- | --- | --- | --- | --- | --- | --- | --- | --- | --- | --- |
|  |  | ⊿𝓧2 | ⊿ df | *P* value | AIC | ⊿𝓧2 | ⊿ df | *P* value | AIC | ⊿𝓧2 | ⊿ df | *P* value | AIC | ⊿𝓧2 | ⊿ df | *P* value | AIC |
| BGdPVS | **ACE** |  |  |  | 424.62 |  |  |  | 412.23 |  |  |  | 392.33 |  |  |  | 392.42 |
|  | CE | 27.557 | 1 | <0.001 | 450.17 | 24.569 | 1 | <0.001 | 434.80 | 21.267 | 1 | <0.001 | 411.60 | 21.285 | 1 | <0.001 | 411.70 |
|  | AE | 0.000 | 1 | 1.000 | 422.62 | 0.000 | 1 | 1.000 | 410.23 | 0.000 | 1 | 1.000 | 390.33 | 0.000 | 1 | 1.000 | 390.42 |
|  | E | 99.472 | 2 | <0.001 | 520.09 | 99.035 | 2 | <0.001 | 507.27 | 82.722 | 2 | <0.001 | 471.05 | 82.630 | 2 | <0.001 | 471.05 |
| WMdPVS | **ACE** |  |  |  | 282.44 |  |  |  | 204.65 |  |  |  | 236.57 |  |  |  | 203.79 |
|  | CE | 123.834 | 1 | <0.001 | 404.27 | 101.976 | 1 | <0.001 | 304.62 | 106.955 | 1 | <0.001 | 341.53 | 102.038 | 1 | <0.001 | 303.83 |
|  | AE | 0.000 | 1 | 1.000 | 280.44 | 0.000 | 1 | 1.000 | 202.65 | 0.000 | 1 | 1.000 | 234.57 | 0.000 | 1 | 1.000 | 201.79 |
|  | E | 254.347 | 2 | <0.001 | 532.78 | 223.451 | 2 | <0.001 | 424.10 | 233.807 | 2 | <0.001 | 466.38 | 223.988 | 2 | <0.001 | 423.78 |

^†^Adjusted for age and sex

^‡^Adjusted for age, sex, volumes of WM for WMdPVS, and BG for BGdPVS

^§^Adjusted for age, sex, and intracranial volume

^¶^Adjusted for age, sex, intracranial volume, volumes of WM for WMdPVS, and BG for BGdPVS

⊿𝓧^2^=chi-square difference, ⊿ df=difference in degree of freedom, AIC=Akaike Information Criterion

**Supporting Table S10.** Comparison of ACE with AE, CE, and E models in bivariate heritability analysis

|  | Model | BG volume | | | | ICV | | | | Pulse pressure | | | | WMdPVS | | | |
| --- | --- | --- | --- | --- | --- | --- | --- | --- | --- | --- | --- | --- | --- | --- | --- | --- | --- |
|  |  | ⊿𝓧^2^ | ⊿ df | *P* value | AIC | ⊿𝓧^2^ | ⊿ df | *P* value | AIC | ⊿𝓧^2^ | ⊿ df | *P* value | AIC | ⊿𝓧^2^ | ⊿ df | *P* value | AIC |
| BGdPVS | **ACE** |  |  |  | 578.84 |  |  |  | 426.32 |  |  |  | 988.15 |  |  |  | 647.60 |
|  | CE | 78.726 | 3 | <0.001 | 651.57 | 83.168 | 3 | <0.001 | 503.49 | 28.950 | 3 | <0.001 | 1011.10 | 155.856 | 3 | <0.001 | 797.46 |
|  | AE | 7.651 | 3 | 0.054 | 580.49 | 1.163 | 3 | 0.762 | 421.49 | 0.000 | 3 | 1.000 | 982.15 | 0.000 | 3 | 1.000 | 641.60 |
|  | E | 356.867 | 6 | <0.001 | 923.71 | 315.583 | 6 | <0.001 | 729.91 | 108.059 | 6 | <0.001 | 1084.20 | 366.629 | 6 | <0.001 | 1002.23 |
|  | Model | WM volume | | | | ICV | | | | Pulse pressure | | | | BGdPVS | | | |
|  |  | ⊿𝓧^2^ | ⊿ df | *P* value | AIC | ⊿𝓧^2^ | ⊿ df | *P* value | AIC | ⊿𝓧^2^ | ⊿ df | *P* value | AIC | ⊿𝓧^2^ | ⊿ df | *P* value | AIC |
| WMdPVS | **ACE** |  |  |  | 179.02 |  |  |  | 259.31 |  |  |  | 834.86 |  |  |  | 647.60 |
|  | CE | 452.432 | 3 | <0.001 | 625.45 | 181.703 | 3 | <0.001 | 435.0 | 133.089 | 3 | <0.001 | 961.95 | 155.856 | 3 | <0.001 | 797.46 |
|  | AE | 0.000 | 3 | 1.000 | 173.02 | 1.511 | 3 | 0.680 | 254.82 | 0.522 | 3 | 0.914 | 829.38 | 0.000 | 3 | 1.000 | 641.60 |
|  | E | 599.697 | 6 | <0.001 | 766.72 | 477.925 | 6 | <0.001 | 725.23 | 272.698 | 6 | <0.001 | 1095.56 | 366.629 | 6 | <0.001 | 1002.23 |

⊿𝓧^2^=chi-square difference, ⊿ df=difference in degree of freedom, AIC=Akaike Information Criterion
